# A Long-Term Macroecological Analysis of the Recovery of a Waterbird Metacommunity after Site Protection

**DOI:** 10.1371/journal.pone.0105202

**Published:** 2014-08-18

**Authors:** Janina Pagel, Alejandro Martínez-Abraín, Juan Antonio Gómez, Juan Jiménez, Daniel Oro

**Affiliations:** 1 Population Ecology Group, Mediterranean Institute for Advanced Studies (Consejo Superior de Investigaciones Científicas-Universitat de les Illes Balears), Esporles, Mallorca, Spain; 2 University of Applied Sciences Bremen, Bremen, Germany; 3 Universidade da Coruña, Departamento de Bioloxía Animal, Bioloxía Vexetal e Ecoloxía, Facultade de Ciencias, A Coruña, Spain; 4 Servicio de Vida Silvestre, Generalitat Valenciana, Conselleria de Infraestructuras, Territori i Medi Ambient, Ciutat Administrativa 9 d′ Octubre, Torre 1, Valencia, Spain; Liverpool John Moores University, United Kingdom

## Abstract

We used the so called “land-bridge island” or “nested-subsets” theory to test the resilience of a highly fragmented and perturbated waterbird metacommunity, after legal protection of 18 wetlands in the western Mediterranean. Sites were monitored during 28 years and two seasons per year. The metacommunity was composed by 44 species during breeding and 67 species during wintering, including shorebirds, ducks, herons, gulls and divers (Podicipedidae). We identified a strong nested pattern. Consistent with the fact that the study system was to a large extent a spatial biogeographical continuous for thousands of years, fragmented only during the last centuries due to human activities. Non-random selective extinction was the most likely historical process creating the nested pattern, operated by the differential carrying capacity (surface-area) of the remaining sites. We also found a positive temporal trend in nestedness and a decreasing trend in species turnover among sites (β-diversity), indicating that sites are increasingly more alike to each other (i.e. increased biotic homogenization). This decreasing trend in β-diversity was explained by an increasing trend in local (α) diversity by range expansion of half the study species. Regional (γ) diversity also increased over time, indicating that colonization from outside the study system also occurred. Overall our results suggest that the study metacommunity is recovering from historical anthropogenic perturbations, showing a high long-term resilience, as expected for highly vagile waterbirds. However, not all waterbird groups contributed equally to the recovery, with most breeding shorebird species and most wintering duck species showing no geographical expansion.

## Introduction

The idea of nestedness in biogeography dates back to the 1930s, but the modern use of the concept to explain diversity patterns in animal communities and metacommunities starts with Patterson and Atmar [1], when studying the community structure of non-volant mammalian faunas in naturally-fragmented archipelagos in montane habitats of the American Rocky Mountains. This concept of nested patterns has been widely applied to landbridge islands [2], [3], those located on the continental shelf, but also to other types of habitats which originally formed a continuous unit to become later on fragmented and isolated due to a variety of reasons, such as mountain tops [4], [5], boreal forests affected by glaciations [6], cloud forest fragments [7], national parks [8] or lake islands [9]. Applied in its original biogeographical sense, a nested pattern means that the species composition of species-poor assemblages is a subset of the species composition of richer assemblages [10], [11]. Many coastal Mediterranean wetlands are also examples of habitats relatively recently isolated and perturbated by anthropogenic activities, which have not received much attention from the macroecological and biogeographical perspectives: nested-subsets or landbridge island framework. Some exceptions are the studies by Paracuellos and Tellería [12] and Sebastián-González et al. [13], who found substantial nestedness in all seasons and years. Lately the old biogeographical concept of nestedness has been adopted to analyze the architecture of mutualistic networks (see e.g. [14]), but that application has nothing to do with the aims of our study.

Here we analyze longitudinally the diversity pattern of a waterbird metacommunity occupying formerly much more continuous, but currently highly fragmented, Mediterranean costal areas to test if it is nested, as expected according to the so-called “landbridge island” paradigm, and also if nestedness has increased over time (see e.g. [15], [16]). This would be expected for a highly vagile zoological group such as waterbirds, and will suggest long-term metacommunity tendency to restore its original structure of spatial homogeneity in species composition once sources of perturbation are under control (i.e. high metacommunity resilience). An increasing trend in nestedness over time should be paralleled by a decreasing trend in species turnover rate (the so-called β-diversity) among sites, since sites become more similar among them.

After approaching the long-term pattern of nestedness we will move on to explore potential (non-random) ecological processes behind it by relating nestedness with either selective colonization or selective extinction [10], [11], [17], accounting as well for other possible causes of nestedness such as passive sampling or habitat heterogeneity [6], [11], [18], [19]. If the pattern is nested we would expect that differential extinction, rather than differential colonization, is the major process creating the nested-subset pattern, because the original situation was one in which all wetlands roughly formed a geographical continuous which has been fragmented by human activities during the last centuries [20]. Reduction in patch size, due to anthropogenic intervention, is known to reduce faunal diversity by shrinking population size leading species to local extinction and lack of colonization [3], [21]. Additionally, selective extinction could be due, in a non-mutually exclusive way, to the losing of habitat types as patch size decreases, affecting more strongly to specialist species [11]. Finally we analyse the applied benefits and drawbacks of having a nested architecture for the long-term resistance and resilience of the system to human perturbations widening the link between nestedness and conservation biology [22], [23], [24], [25].

## Methods

### The data set and field procedures

**Figure 1:**
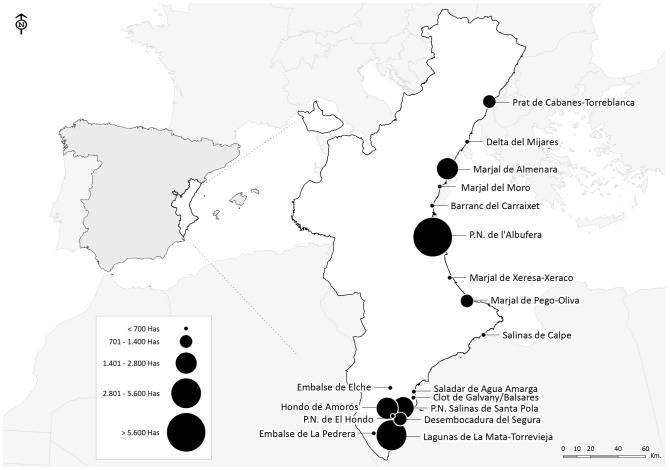
Location and size of the study wetlands in Eastern Spain.

An official data set on bird counts for a 28-year period (1984–2011), compiled over the years by the environmental authority of Comunidad Valenciana region (i.e. Generalitat Valenciana), was used to analyse the nested pattern of a waterbird metacommunity including 44 breeding and 67 wintering species in 18 wetlands in the western Mediterranean (Eastern Spain). We show in Figure 1 the location of sites, their relative size and distance among them. To the best of our knowledge probabilities of species detection can be considered to be constant across sites and seasons from year to year as both methodology and human team composition have remained approximately constant during the study period, and hence results derived from species-richness data are comparable among years despite the biases that differential detectability among species could introduce in the absolute estimates of our metrics of nestedness [18].

No birds were collected or samples taken. Two of the co-authors (J. A. Gómez and J. Jiménez) are the civil servants from the regional government in charge of coordinating field teams, and the authors have collaborated directly on the detection and count of waterbirds in the main study sites over many years. Our field work did not violate any law or invaded private land at all. Many sites in this study are protected as nature parks (i.e. Cabanes, Albufera, Pego-Oliva, Santa-Pola, Torrevieja, El Hondo) since 1986–1988 or have been protected afterwards as Important Bird Areas (IBA) (2007–2009) by the regional environmental authority. Detailed information of the sites protected as IBAs can be consulted at: http://www.docv.gva.es/datos/2009/06/09/pdf/2009_6699.pdf.

Winter counts were performed simultaneously in all wetlands each year during two weeks around the second weekend of January, in coordination with the International Waterbird Census (IWC) (for further details see http://www.wetlands.org/AfricanEurasianWaterbirdCensus/tabid/2788/Default.aspx). Wintering ducks, coots or divers (Podicipedidae) were counted from the distance and from fixed sites every year using scopes. Other wintering bird groups such as herons, gulls or shorebirds were counted along fixed car itineraries with variable detection band widths depending on the characteristics of each study site. Wintering marsh harriers were counted around sunset at communal roosts.

Breeding season counts were not coordinated internationally and were mostly carried out by the staff of protected areas that monitor study sites. Visits to the study area were carried out almost on a daily basis over the whole breeding season (March-August) to prevent overlooking relevant information due to the lack of complete overlap in the breeding calendar of the study species. Counts were performed using specific and fixed methodologies for each species. Colonial species (herons, gulls, terns, shorebirds, and flamingo) were counted by visiting breeding colonies and counting individual nests at the peak of their breeding period. Non-colonial species (ducks, coots, Podicipedidae) were detected by inspecting water masses by means of motor boats, counting nests or birds displaying breeding behaviour or adults in the company of chicks. Species of difficult detection (rallid species, little bittern) were detected prospecting the study area in detail by means of boats propelled manually in shallow water areas. Further information on winter and summer counts for the whole study region during the period 1984–2004 is available at http://www.cma.gva.es/webdoc/documento.ashx?id=164402.

Most study sites were former (Holocene) coastal lagoons in different stages of its natural succession towards terrestrial ecosystems, but with a high degree of human influence on all components of the structure of their animal and plant communities. Evidence for this is the high rate of loss of suitable habitat in Mediterranean coastal wetlands for many zoological groups, including birds, reported during the decades prior to habitat protection (see e.g. [26], [27]).

### Overall nestedness

For the nestedness analysis we first assembled presence-absence summary matrices from the dataset of bird counts obtained as described above, both for breeding and wintering seasons, with wetlands in rows and bird species in columns. A presence hence indicates that the species has been observed in a particular wetland at least once during the period 1984–2011. To quantify the degree of nestedness for the summarized qualitative matrices we calculated two metrics, a) the matrix temperature (T) [21] and b) the nested overlap decreasing fill (NODF) [28] with software ANINHADO 3.0 [28], [29]. Specifically ANINHADO uses the dispersal of unexpected presences or absences in the maximally packed matrix to derive the observed temperature of the matrix which varies from 0° (perfectly nested) to 100° [28]. The NODF metric is based on standardized differences in row and column fills and paired matching occurrences, ranging from 100 (perfectly nested) to 0 [11]. The observed temperature or NODF is then compared to the mean temperature of a frequency distribution of one thousand Monte-Carlo simulated temperatures (or NODFs) obtained under a null model selected out of four null models available. We used null model number two (CE) because it calculates the probability, that a cell (a_ij_) in the simulated matrix shows a presence, as 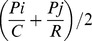 where P_i_ =  number of presences in the row i; P_j_ =  number of presences in the column j; C =  number of columns; R =  number of rows, that is the assignment of a presence takes into account data-derived information (i.e. species distribution range and wetland richness), whereas all other three null models available do the assignment of presences either by columns only, rows only or at random. Hence results derived from null model 2 could be considered more restrictive. The species that are present in most wetlands are placed in the top left column, whereas wetlands with the highest number of species are placed in the topmost row. ANINHADO orders wetlands so that nestedness is maximally visualized. A perfectly nested system has a 50% fill in the upper-left corner of the packed matrix [28]. To visualize the maximally packed matrix we used the “bipartite” package in R software [30]. In this graphical representation of the geographical matrix black squares stand for species presences and white squares for species absences. We chose to use both metrics to a) allow comparisons with previous studies based on temperature, and b) provide information regarding the suitability of one of the metrics over the other by checking whether results from both metrics coincided or not.

To compare the degree of nestedness of qualitative matrices with that of quantitative matrices we assembled quantitative matrices with the average of abundance of each species in each wetland (wetlands in rows, species in columns) both for breeding and wintering for the period 1984–2011. We used software NODF (not to confuse with the NODF metric used together with qualitative matrices) [31] to calculate the degree of nestedness of the quantitative matrices. A nested pattern with a quantitative matrix, compared to a qualitative matrix, not only means that the species composition in smaller assemblages is a subset of that in larger assemblages but that their abundances are also nested (i.e. all populations making up local assemblages have lower abundances than their conspecific populations in richer assemblages) [31]. To use NODF software the unpacked quantitative matrices were modified first by software EcoSim 7.0 [31], [32]. EcoSim changes the format of the quantitative matrix in a space delimited text file matrix after importing the quantitative matrix from Microsoft Excel. This text file is needed to run NODF software. To calculate the degree of nestedness using quantitative data NODF software uses a modification of the NODF index [28] called Weighted Nestedness metric based on Overlap and Decreasing Fill (WNODF). WNODF measures the degree of nestedness based directly on overlap and decreasing fill, and, in contrast to temperature, with a range of degree of nestedness from 100 (perfectly nested) to 0 [31]. To measure the degree of nestedness WNODF quantifies if the marginal total (i.e. incidences or richness) of a given sequence of columns or rows decrease and also if the study system loses species in an ordered way, as in the case of NODF or T. We used null model rc that assigns individuals to matrix cells proportionally to observed row and column abundance totals until, for each row and column, total abundances are reached [31]. As sorting option we used row/column abundance totals.

To verify the degree of nestedness of our maximally packed qualitative and quantitative matrices and to compare them with other studies we calculated standardized effect sizes, which measure the number of standard deviations that the observed index is above or below the mean index of the simulated index [13], [33]. For temperature (T) of the qualitative matrix we calculated the standardized effect size as a z-score (observed T – mean simulated T)/standard deviation of the simulated T; for NODF we obtained a relative NODF (observed NODF – mean simulated NODF)/mean simulated NODF following Montesinos-Navarro et al. [34]. For the quantitative matrix we calculated the standardized effect size (z′-score) with NODF software in the same way as the z-score for the qualitative matrix is calculated. A z-score with a value below -2.0 or above 2.0 indicates approximate statistical significance for α at the 5% a priori risk level of committing a Type I error [13], [35]. The relative NODF values cannot be compared directly with temperature values.

We ordered the wetlands of the overall qualitative matrices both for breeding and wintering by their degree of nestedness calculated with BINMATNEST [36]. BINMATNEST reorders rows and columns until nestedness is maximized and unexpectedness is minimized by using a genetic algorithm that is more accurate to order rows and columns than that used by other programs [15], [36]. We explored whether selective extinction or selective colonization were the processes behind the nested pattern by calculating Spearman rank correlation coefficients. We correlated the row order of the qualitative packed matrix with the size of each wetland (extinction), and also with the distance to the nearest wetland (colonization) using the R software [30]. We verified whether results were similar when data were not subjected previously to BINMATNEST.

### Inter-annual variability in nestedness and diversity

To calculate the degree of inter-annual variability in nestedness we used the same software and calculations as to obtain the degree of overall nestedness, but for each year of the study period both for breeding and wintering, using all three standardized effect sizes. Since we detected that the three effect sizes used showed, as a rule, an increasing trend over time, we fitted general linear models to the observed trend in order to determine their strength (slope), statistical significance (95% confidence interval) and degree of fit (r^2^). In order to find out whether increasing degree of nestedness was paralleled by a decrease in beta-diversity we calculated β-diversity both for breeding and wintering as 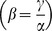, where γ is gamma or regional diversity (i.e. the number of species in all our metacommunity) and α is local diversity (i.e. the arithmetic mean of the number of species in each of our study sites) [37]. To analyze the trends in β, α and γ-diversity we also fitted general linear models to data in R. Given that during the years from 1984 to 1989 a smaller sampling effort was done (i.e. a smaller number of wetlands were censused) we used the time series only from 1990 on, to analyze the trend in nestedness over time. Range expansion was calculated by subtracting the average of the number of occupied sites in the second half of the time series (2001–2011) from the average number of occupied sites during the first half of it (1990–2000) (“Δsites” hereon). The second half of the time series corresponds approximately to a period of consolidated protection of the sites, after a decade of protection by law as nature parks, chosen so that sample size is equalized with the first half of the time series.

## Results

### Overall nestedness

**Figure 2:**
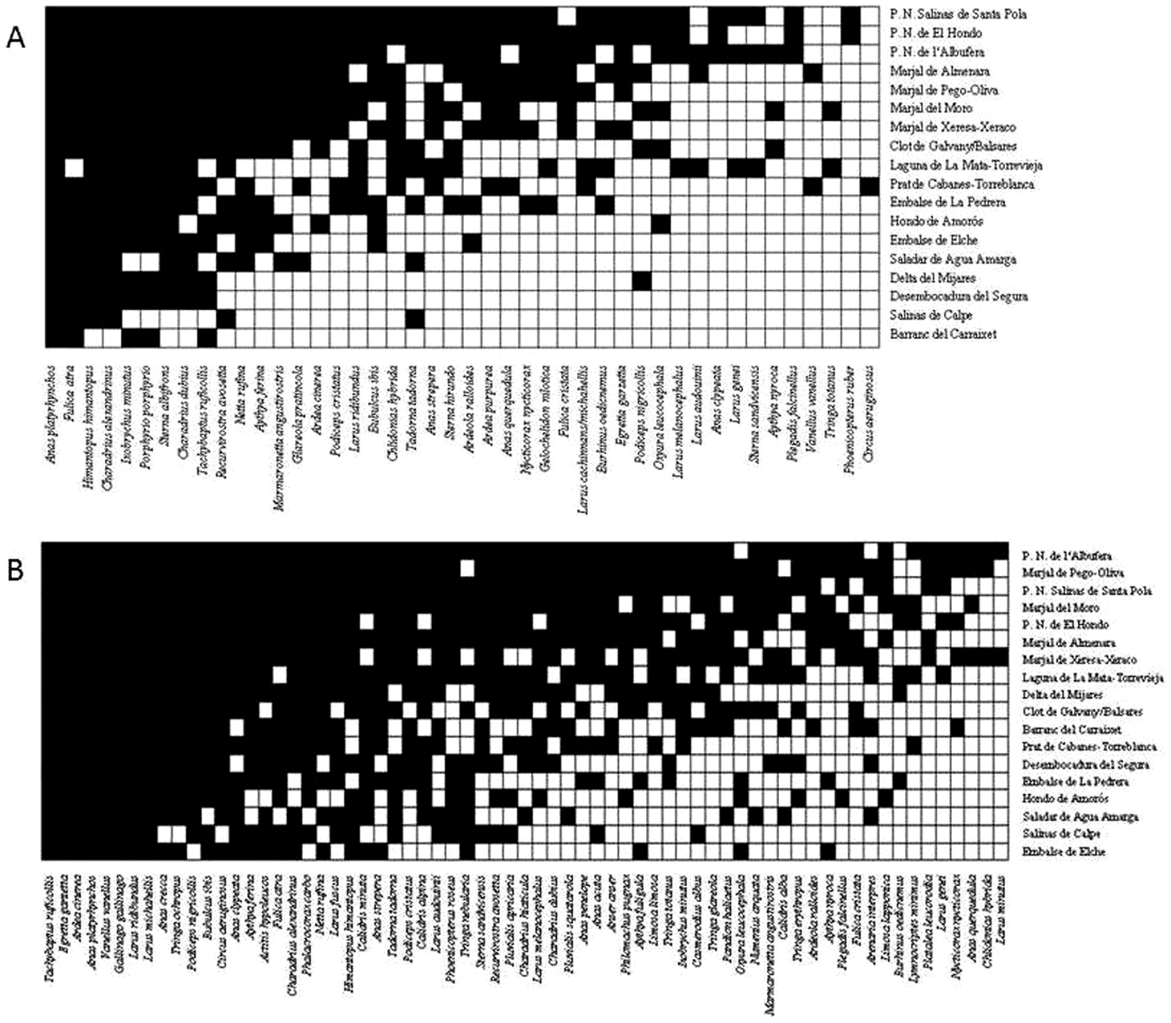
Overall nestedness of the waterbird metacommunity. Graphical representation of the qualitative maximally packed matrix during A) the breeding season and B) the wintering season (1984–2011). Black dots are species presences recorded at least once during the study period; white dots are species absences. A perfectly nested system would have a 50% fill in the upper-left corner.

The waterbird metacommunity was found to be highly nested, both during the breeding and wintering seasons, as observed temperatures were quite low (Figure 2, Table 1). The qualitative matrix showed a higher nested pattern during breeding than during wintering, as determined by a higher (negative) z-score during breeding. Higher nestedness during the breeding period was also detected when using the relative NODF metric with the qualitative matrix. The values of the standardized effect sizes for WNODF in the quantitative matrix indicated no nestedness either during wintering or breeding (Table 1 B).

**Table 1:** Analysis of the overall nestedness of the waterbird metacommunity studied during the breeding and wintering seasons.

	Qualitative matrix										
	Temperature (T)						NODF				
Season	Observed	Simulated	SD	Lower 95% CI	Upper 95% CI	z	Observed	Simulated	Lower 95%/CI	Upper 95%/CI	NODF_r_
Breeding	12.32°	46.11°	3.65	38.96°	53.26°	−9.26	77.75	56.99	52.72	61.26	0.36
Wintering	18.21°	48.30°	3.59	41.26°	55.34°	−8.38	80.89	69.73	66.83	72.63	0.16

	Quantitative matrix										
	WNODF										
Season	Observed	Simulated	SD	Lower 95% CI	Upper 95% CI	z'					
Breeding	35.24	62.38	2.15	58.04	66.15	−12.65					
Wintering	50.22	69.27	1.67	65.97	72.44	−11.42					

Qualitative Matrix: the values are based on the overall qualitative matrix (a species presence or absence in a site within 28 years). Simulated T/NODF is in each case the average of 1000 Monte Carlo simulations run in ANINHADO. SD  =  standard deviation of simulated T. CI = 95% confidence interval of simulated T/NODF. Z  =  standardized effect size of T (see text). NODF_r_  =  Relative NODF (see text).

Quantitative Matrix: the values are based on the overall quantitative matrix (an average of species abundance in a site within 28 years). Simulated WNODF is in each case the average of 1000 Monte Carlo simulations run with the NODF software. SD  =  standard deviation of simulated WNODF. CI = 95% confidence interval of simulated WNODF. Z'  =  standardized effect size of WNODF (see text).

### Temporal dynamics in nestedness

**Figure 3:**
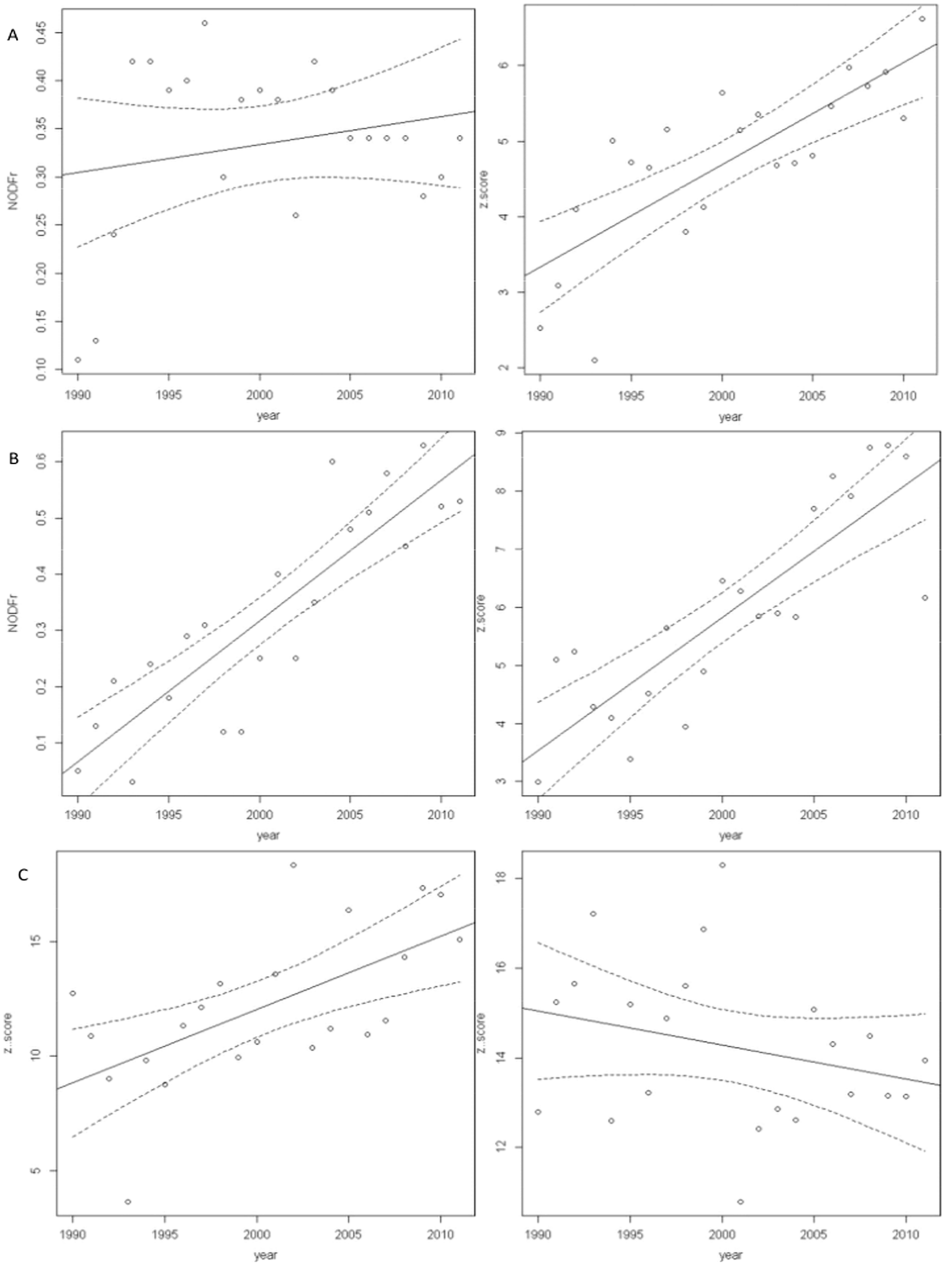
Inter-annual variability of degree of nestedness of the qualitative and quantitative matrices during the breeding and wintering seasons (1990–2011). This figure shows the inter-annual variability of degree of nestedness of the qualitative matrix during A) breeding season, B) wintering season and of the C) quantitative matrix (left breeding, right wintering). Note that absolute values of z′-scores were used despite z-values are negative. z. =  z-score; z. =  z′-score. The lines show the best fit (solid line) and 95% confidence bands (dotted line). See Table 2 for a summary of parameter estimates of the general linear models fitted to these standardized effect sizes.

**Figure 4:**
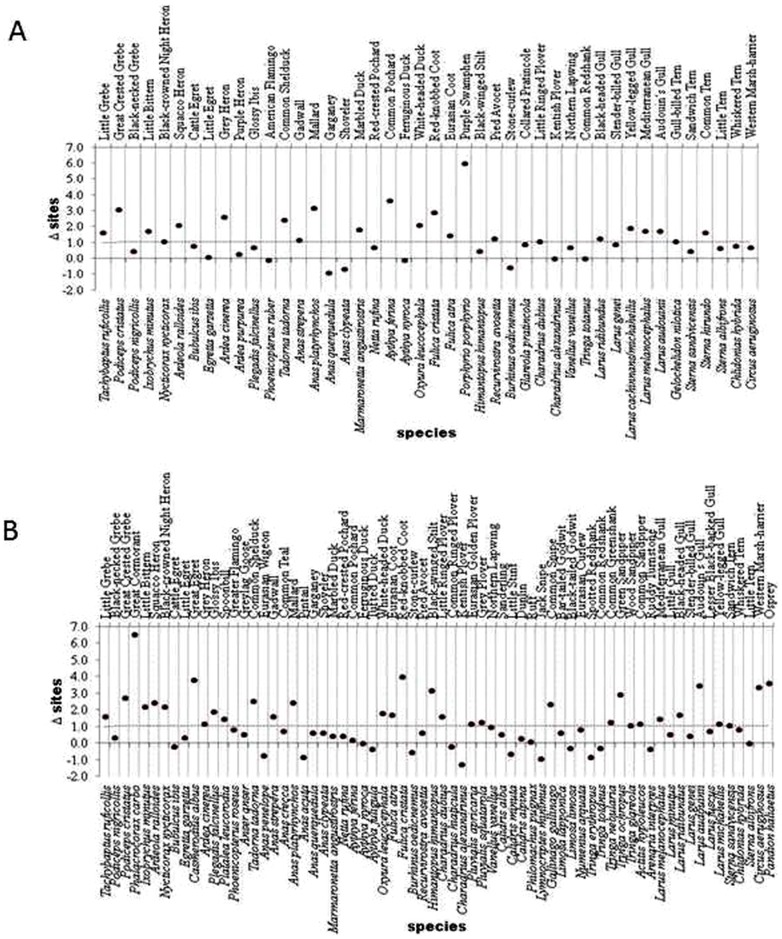
Delta-sites or change in number of sites occupied by each study species over time (1990–2011). Delta-sites are shown for both A) breeding and B) wintering. Delta-sites is defined as the subtraction of the average of the number of occupied sites in the second half of the time series (2001–2011) from the average number of occupied sites during the first half of the time series (1990–2000). Positive values of the index indicate species expansion. The dotted line is the arbitrary minimum value beyond which we consider range expansion is taking place.

The study metacommunity showed a nested pattern in most study years, when using the qualitative but not when using the quantitative matrix (Table S1). Interestingly, the z-scores of the qualitative matrix showed a strong positive trend over time (Figure 3, Table 2), indicating increasing nestedness during both study seasons, contrarily to that found by other authors for artificial Mediterranean wetlands [13]. The values of NODF_r_ also showed a strong positive trend during wintering, although not during breeding (Figure 3, Table 2). Regarding the quantitative matrix the increasing trend of the negative values of z′-scores during breeding indicated poorer nestedness with time (i.e. smaller observed values of the metric compared to simulations). During wintering no pattern of nestedness appeared for the quantitative matrix (Figure 3, Table 2). Regarding Δsites results indicated that roughly 50% of the species, both in wintering and summer, were expanding their ranges (Figure 4).

**Table 2:** General linear models fitted to the change of the standardized effect sizes over time both for the breeding and wintering seasons and for the qualitative and quantitative matrices.

Matrix	Season	Metric	Slope	Lower 95% CI	Upper 95% CI	r^2^
Qualitative	Breeding	NODF_r_	0.0029	−0.0030	0.0088	−0.0031
		z-score	**0.1353**	0.0891	0.1815	0.6031
	Wintering	NODF_r_	**0.0251**	0.0189	0.0312	0.7482
		z-score	**0.2291**	0.1648	0.2934	0.6946
Quantitative	Breeding	z‘-score	**0.3211**	0.1418	0.5003	0.0022
	Wintering	z‘-score	−0.0762	−0.1935	0.0412	0.0286

CI = 95% confidence interval of the slope. r^2^  =  coefficient of determination. NODF_r_  =  Relative NODF. Z  =  standardized effect size of temperature. Z'  =  standardized effect size of WNODF. Values in bold are statistically significant (i.e. 0 is within the 95% confidence intervals).

### Beta-diversity and nestedness

**Figure 5:**
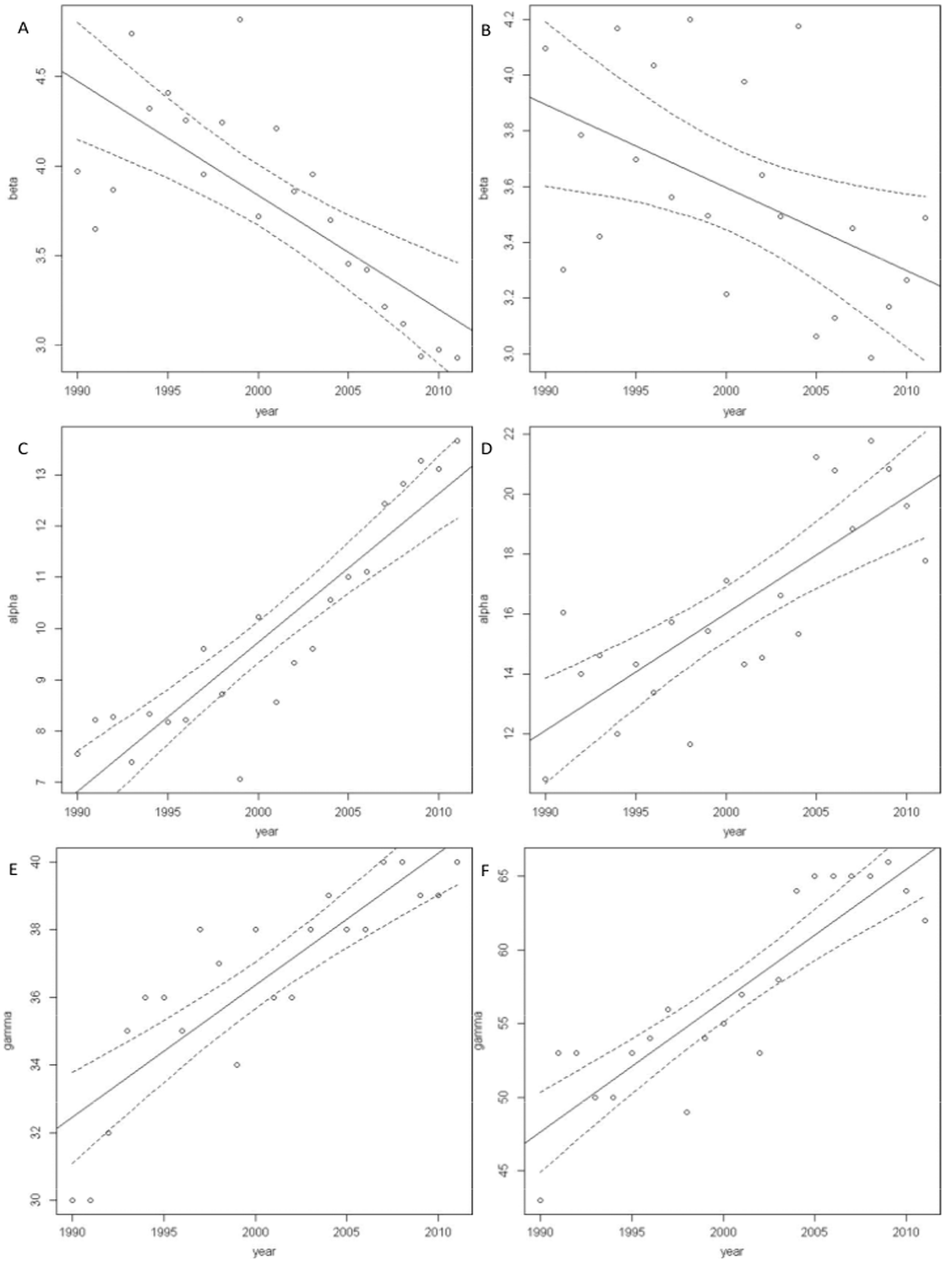
Beta-, alpha- and gamma-diversity over time (1990–2011). Trend of β-diversity over time for A) breeding and B) wintering; α-diversity over time for C) breeding and D) wintering, and γ-diversity over time for E) breeding and F) wintering. Solid lines are the lines of best fit and dotted lines are the 95% confidence intervals.

In order to validate our results on increased nestedness over time we calculated beta-diversity. As nestedness increases β-diversity is expected to decrease in a closed system [38], [39], [40]. However, we found a decreasing trend in β-diversity in our open system during breeding (slope = −0.06; 95% CI slope = −0.09, −0.04; r^2^ = 0.53) (Figure 5) and wintering (slope = −0.03; 95% CI slope = −0.05, −0.01; r^2^ = 0.21) (Figure 5). Since β-diversity is calculated as gamma-diversity over alpha-diversity a decrease in β-diversity can be due either to a decrease in gamma-diversity or to an increase in alpha-diversity [41], [42]. We found indeed an increasing trend in α-diversity during breeding (slope = 0.29; 95% CI slope = 0.23, 0.35; r^2^ = 0.81) (Figure 5) and, wintering (slope = 0.39; 95% CI slope = 0.26, 0.52; r^2^ = 0.6) (Figure 5). But interestingly, we also found strong increasing trends in γ-diversity during wintering (slope = 0.89; 95% CI slope = 0.68, 1.1; r^2^ = 0.77) and breeding (slope = 0.39; 95% CI slope = 0.29, 0.49; r^2^ = 0.72;) (Figure 5). Spearman rank correlations between β-diversity and nestedness were strong and statistically significant when using the z-score both for breeding (r_s_ = −0.69; p<0.001) and wintering (r_s_ = −0.74; p<0.001), although not when using the NODF_r_ metric.

### Extinction versus colonization processes

Spearman rank correlation coefficients between row order of the packed qualitative matrix and size of the wetlands were negative, strong, and statistically significant, in most years and in both seasons (Table 3, Table S2), either using or not BINMATNEST to re-order rows and columns, suggesting a role of selective extinction in creating the nestedness pattern observed (i.e. nestedness generated by ordered species loss). On the contrary, the Spearman rank correlation coefficients between row order and distance to the nearest wetland were both positive and negative, showed very low values and, as a rule, were not statistically significant (Table 3), suggesting a low influence of selective colonization on the observed pattern of nestedness.

**Table 3:** Spearman rank correlation coefficients (r_s_) for the summary matrices and annual variability of correlations between row order for the matrices packed by BINMATNEST, and wetland size as well as distance to the nearest wetland.

Year	Breeding season				Wintering season			
	Wetland size (ha)		Distance to the nearest wetland (km)		Wetland size (ha)		Distance to the nearest wetland (km)	
	Coefficient	n	Coefficient	n	Coefficient	n	Coefficient	n
Summary matrix	**−0.767**		0.068		**−0.769**		−0.236	
1984					**−0.841**	13	−0.290	13
1985	**−0.655**	11	0.123	11	**−0.670**	13	0.256	13
1986	−0.370	10	0.268	10				
1987	−0.309	10	−0.067	10	−0.418	10	0.267	10
1988	**−0.776**	12	0.270	12	**−0.745**	10	0.256	10
1989	**−0.764**	11	0.342	11	**−0.687**	13	0.405	13
1990	**−0.621**	16	**−0.001**	16	**−0.664**	15	0.081	15
1991	**−0.603**	16	0.133	16	**−0.652**	17	−0.129	17
1992	**−0.650**	16	0.261	16	**−0.716**	17	−0.716	17
1993	−0.564	10	0.482	10	**−0.650**	17	−0.219	17
1994	**−0.611**	15	−0.419	15	**−0.688**	14	0.156	14
1995	**−0.837**	14	−0.119	14	**−0.723**	14	**0.024**	14
1996	**−0.850**	15	−0.136	15	**−0.824**	13	−0.094	13
1997	**−0.682**	16	−0.109	16	**−0.809**	16	0.077	16
1998	**−0.775**	15	**−0.030**	15	**−0.903**	16	−0.215	16
1999	−0.370	10	**0.049**	10	**−0.771**	16	−0.071	16
2000	**−0.679**	15	**0.030**	15	**−0.850**	17	−0.157	17
2001	**−0.631**	14	**−0.035**	14	**−0.647**	16	−0.192	16
2002	**−0.560**	16	−0.123	14	−0.121	15	−0.192	15
2003	−0.451	13	**−0.036**	13	−0.534	14	**−0.002**	14
2004	−0.544	13	0.339	13	**−0.684**	14	0.064	14
2005	**−0.648**	13	0.196	13	**−0.718**	16	−0.158	16
2006	**−0.679**	14	**0.040**	14	**−0.644**	16	−0.141	16
2007	**−0.857**	15	0.147	15	**−0.600**	15	−0.086	15
2008	**−0.811**	15	0.116	15	**−0.543**	15	−0.211	15
2009	**−0.746**	15	0.154	15	**−0.578**	17	−0.264	17
2010	**−0.888**	16	**−0.007**	16	**−0.521**	16	0.077	16
2011	**−0.777**	18	−0.083	18	**−0.885**	16	0.069	16

We show in bold the statistically significant results. N = Number of rows which were used for the correlation (for the overall matrices 18 rows were used for all correlations). No data provided in the breeding season of 1984 and wintering season of 1986 for the qualitative matrix because less than 10 rows of those ordered matrices contained data.

## Discussion

Qualitative matrices showed that the waterbird metacommunity was highly nested, both during the wintering and breeding seasons, but nestedness was higher during the breeding season. The metacommunity also showed increasing trends of nestedness over time, both during wintering and breeding. Nestedness however was not found when using quantitative matrices. The difference between qualitative and quantitative results suggests that compositional and density changes do not follow the same structural rules. Wetlands can recover lost species but not necessarily their abundances need to remain lower in the wetland gaining the species, compared to the donating wetland. Hence presence/absence has little to do with densities. Compared to the results for the waterbird community in completely artificial wetlands (i.e. irrigation ponds) [13] our negative z-scores were higher indicating a higher nestedness of our study system involving mostly natural wetlands or former natural wetlands transformed by human action such as salt-pans.

### Processes generating nestedness

According to some authors [11] a high nestedness in a metacommunity can be the result of several causes. False negatives in the qualitative matrix can be due to imperfect detection since nestedness studies typically do not account for detection probability [18]. These false zeros would reduce the degree of nestedness incorrectly. In our case sampling artefacts are very implausible because we have used a long-term (28-years) time series for our study, and hence it is very unlikely that our summarized matrix is missing some species which are present but not detected in the study system. Sampling artefacts are not to be mistaken with the mechanism of passive sampling [11], [43]; in this case, species colonize fragmented habitats proportional to their abundance. In our study system some abundant species such as herons were lost as we moved from bigger to smaller wetlands, especially during breeding, suggesting that factors other than passive sampling, related to size of the wetland or habitat heterogeneity, were most likely acting.

Differences in water quality probably did not influence the degree of nestedness of the metacommunity [6], because more or less similar efforts have been devoted to water quality restoration in all wetlands. Hence we have presently an array of wetlands in which most sites are all in a similar (although still poor) water quality condition. One particular component of habitat quality is human disturbance. Some authors have found that nestedness can be promoted by human disturbance but depending on its level and the disturbance tolerance of the species [19]. In our case differences in human disturbance are not likely a cause of nestedness because most coastal wetlands in the study region are effectively protected as nature parks, and human uses are alike.

A further option to get a nested pattern is to have an array of sites with different habitat heterogeneity so that habitat type is nested in the sense that sites with smaller species assemblages have a subset of the habitats present in the richer sites. Losing habitats sequentially can lead to losing species in an ordered way [11]. According to our long experience in the study area, that factor is most likely influencing nestedness, but we have no fine-grain quantitative data available to test its influence. However habitat heterogeneity is most likely highly correlated with wetland size (area) and probably the identified influence of decreasing wetland size on the loosing of species is in fact driven by the loss of habitat heterogeneity [44]. An alternative causal factor of nestedness is the fact that the loss of species may be proportional to local abundance (i.e. population size or density).

### Selective extinction and selective colonization

The theory of island biogeography [45], [46] predicts that a fragmented habitat tends to lose species as its size decreases, and that colonization decreases as a direct function of patch isolation, although there are some exceptions (see e.g. [47]). Our results suggest that selective extinction was the most likely historical cause generating nestedness in our waterbird metacommunity, from the original situation in which the study area roughly formed a continuous to the highly fragmented pattern of today (see [48]). This result is consistent with the findings done by other authors working with waterbirds [13] since they detected pond size-dependent selective extinction as the main cause of nestedness in artificial wetlands. Selective colonization did not play a relevant role in creating nestedness in our study system most likely due to the highly vagile nature of the study group (i.e. birds with a high colonization capacity) (see e.g. [49]). Selective extinction in our study system could be related to two factors, either a) wetlands lose species as they become smaller because population size decreases below the minimum viable population size [9]. This may lead to deterministic Allee effects (i.e. deterministic problems in finding food, mates or defence against predators at low densities). Also the different species could be forced to using similar resources as wetland size decreases, and hence deterministic competitive exclusion among species might take place. Finally, demographic stochasticity could lead to loosing species just by random changes in demography (i.e. random changes in vital rates such as fecundity or survival) as wetland size and, in turn, overall population size, shrinks. Or b) wetlands lose habitat heterogeneity (i.e. nested habitat hypothesis) and hence species associated to those habitats. As already-stated we do not have detailed information on habitat type presence and abundance for each study wetland, and thus we cannot rule this factor out. The most vulnerable bird groups to reduction in wetland size were herons, divers and gulls in the breeding season and ducks and shorebirds during wintering (Figure S1).

### Between seasons variability in nestedness

We found a solid difference between the compositional structure of our breeding and wintering communities, with a higher nestedness always taking place during the breeding season, regardless of the metric used (temperature or NODF). This result is coincident with the structure found in artificial wetlands located in the southern tip of our study region [13]. It is likely that the process of selective extinction is affecting more heavily waterbird species during the reproductive season. This may be so because habitat requirements are probably more demanding during breeding than wintering, because of the need of getting resources for both parents and offspring, especially in the Mediterranean region, where the highest temperatures of the annual cycle coincide with the lowest precipitations. Breeding birds need appropriate nesting habitat, quietness and enough food of high quality for their offspring.

### Inter-annual variability in nestedness

We found an increasing trend of nestedness in the waterbird metacommunity over time confirmed through a decrease in β-diversity in both seasons [40]. The main reason why β-diversity decreased was probably by the fact that α-diversity (the local number of species in each wetland) also increased over time due to species reshuffling among sites generating the pattern of increased nestedness with time. Actually β-diversity did not decrease faster due to increased nestedness because we also found an increasing trend in γ-diversity in our open system, that is gaining species from outside by immigration (e.g. Spoonbill, Great Egret, Glossy Ibis). That could shed some light to the current debate on the determinants of β-diversity [50], [51] (but see [52], [53]). Our analysis of the change in number of sites occupied by each species indicated that 50% of the species expanded geographically over the study period (i.e. secondary colonization or/and immigration). Frequent colonization is likely to enhance nestedness [23], as it reduces the number of unexpected absences.

### Conservation implications

The increase in nestedness over time could be initially interpreted as a negative result from a conservation viewpoint because it means increasing the biotic homogenization of the system (by losing β-diversity) [54]. However, it also has a positive interpretation. By increasing nestedness the system is showing a high resilience to recover from historical fragmentation and perturbation after only two and a half decades of legal site protection. Increased nestedness also leads to gaining overlap among wetland biotas and hence probably to increased resistance and resilience against perturbations, as the system becomes more and more redundant [23]. Thus losing one of the species in a site is not so relevant for the whole metacommunity, as it can be recovered by reshuffling of local species (i.e. secondary colonization). Hence, in summary, we can conclude that our study system is becoming more and more homogenized because of species expansion. These results may suggest that the regional system of protected wetlands studied is showing some positive results, despite the degree of fragmentation has remained approximately unchanged and extensive work remains to be done for the full recovery of water quality and habitat heterogeneity. It is a fact that this system was in a very impoverished state at the beginning of our study period (1980s) according to the rich composition of its avian communities up to the 1970s (see e.g. [55]), and hence we are most likely observing a recovery of the original metacommunity by immigration and also by range expansion of local species, during the last decades, following some improvement in environmental conditions and reduction in human pressure. This recovery of the metacommunity is likely due not only to the local protection of sites, but also to the improving conditions in wetlands outside the study system, at the regional, national and trans-national levels [56], [57], [58], [59]. Additionally, the metacommunity has gained some species by means of reintroduction programmes (i.e. Red-knobbed Coots and Purple Swamphen) and probably due to increasing temperatures at the regional level, because former migrating species during the winter now remain in our study sites; clear examples are Little Tern, Squacco Herons, Black-crowned Night Heron and Black-Winged Stilt. Hence the study system is not any more within the stage of ecological relaxation (i.e. gradual losing of species by increased fragmentation). Obviously, the fact of dealing with a highly vagile animal group makes the recovery of the whole system (covering several hundred kilometres in length) more viable.

However, not all bird groups contributed equally to homogenization (see e.g. [7]). During the breeding season, shorebirds, gulls and herons comprised 29%, 25% and 25% respectively of the species performing poorly in the sense of lack of expansion. In winter shorebirds and ducks represented 43% and 30% of the species not under expansion. Within groups 87% of the shorebird species were not expanding in breeding and 73% of the duck species in winter. This suggests scarcity of suitable breeding habitat during the summer for shorebirds and a poor water quality for wintering ducks, especially for diving species dependent on submerged vegetation. Both of these matters (water quality and habitat heterogeneity) are the key factors to be improved in the near future to allow the full recovery of the former waterbird metacommunity. However, since immigration from outside the system also plays a role, the temporal trends of breeding shorebirds and wintering ducks should be explored at large geographical scales to make sure that the lack of local recovery of these groups is not only due to poor suitability of the study wetlands for them but also to larger-scale problems either in Africa or central and northern Europe (see e.g. [60]).

## Supporting Information

Figure S1 **Loosing species in relation to wetland size reduction.** Species loss by zoological groups in relation to wetland size reduction for both A) breeding and B) wintering season.
(TIF)

Table S1 **Analysis of the annual variability of nestedness of the waterbird metacommunity studied during breeding and wintering.** Qualitative Matrix: simulated T/NODF is in each case the average of 1000 Monte Carlo simulations run in ANINHADO. SD  =  standard deviation of simulated T. CI = 95% confidence interval of simulated T/NODF. Z  =  standardized effect size of T (see text). NODF_r_  =  Relative NODF (see text). Values in bold are statistically significant results (the observed temperature/NODF is not within the 95% confidence intervals). Quantitative Matrix: simulated WNODF is in each case the average of 1000 Monte Carlo simulations run in NODF. SD  =  standard deviation of simulated WNODF. CI = 95% confidence interval of simulated WNODF. Z'  =  standardized effect size of WNODF (see text).
(DOC)

Table S2 **Order of nestedness of the overall qualitative matrix for breeding and wintering season.** The order of nestedness is according to the degree of nestedness packed by BINMATNEST.
(DOCX)
