# Supplementary figures and images for: A Long-Term Macroecological Analysis of the Recovery of a Waterbird Metacommunity after Site Protection
Source: PLoS One. 2014 Aug 18;9(8):e105202. doi: 10.1371/journal.pone.0105202 (PMC4136829; doi:10.1371/journal.pone.0105202)

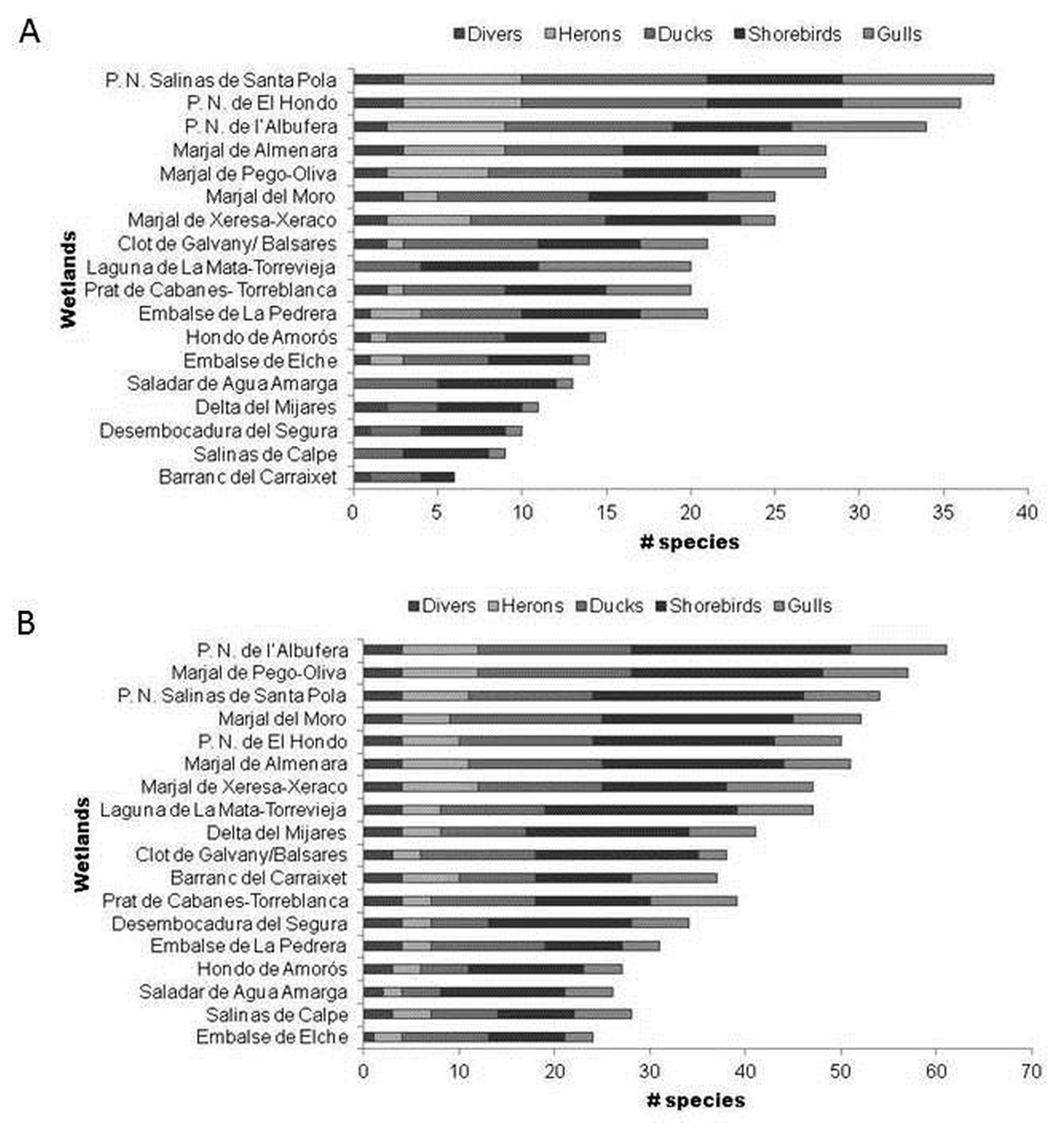

Supplement: Figure S1 — Loosing species in relation to wetland size reduction. Species loss by zoological groups in relation to wetland size reduction for both A) breeding and B) wintering season. (TIF) [file pone.0105202.s001.tif]
