# Supplementary material for: A Long-Term Macroecological Analysis of the Recovery of a Waterbird Metacommunity after Site Protection
Source: PLoS One. 2014 Aug 18;9(8):e105202. doi: 10.1371/journal.pone.0105202 (PMC4136829; doi:10.1371/journal.pone.0105202)
Supplement: Table S1 — Analysis of the annual variability of nestedness of the waterbird metacommunity studied during breeding and wintering. Qualitative Matrix: simulated T/NODF is in each case the average of 1000 Monte Carlo simulations run in ANINHADO. SD = standard deviation of simulated T. CI = 95% confidence interval of simulated T/NODF. Z = standardized effect size of T (see text). NODFr = Relative NODF (see text). Values in bold are statistically significant results (the observed temperature/NODF is not within the 95% confidence intervals). Quantitative Matrix: simulated WNODF is in each case the average of 1000 Monte Carlo simulations run in NODF. SD = standard deviation of simulated WNODF. CI = 95% confidence interval of simulated WNODF. Z' = standardized effect size of WNODF (see text). (DOC) [file pone.0105202.s002.doc]

| Qualitative matrix | | | | | | | | | | | | |
| --- | --- | --- | --- | --- | --- | --- | --- | --- | --- | --- | --- | --- |
| Temperature (T) | | | | | | | | NODF | | | | |
| Year | Season | Observed | Simulated | SD | Lower  95 % CI | Upper  95 % CI | Z | Observed | Simulated | Lower  95 %/ CI | Upper  95 %/ CI | NODF_r_ |
| 1984 | Breeding | 14.78° | 38.54° | 19.04 | 1.22° | 75.86° | -1.25 | 13.40 | 9.76 | 5.06 | 14.46 | 0.37 |
| 1985 | Breeding | 18.89° | 28.93° | 7.36 | 14.50° | 43.36° | -1.36 | 26.58 | 21.31 | 15.37 | 27.25 | 0.25 |
| 1986 | Breeding | 12.63° | 28.31° | 8.52 | 11.61° | 45.01° | -1.84 | **25.89** | **19.52** | **13.23** | **25.81** | **0.33** |
| 1987 | Breeding | **10.81°** | **27.36°** | **8.23** | **11.23°** | **43.49°** | **-2.01** | 23.76 | 19.89 | 13.87 | 25.91 | 0.19 |
| 1988 | Breeding | **14.86°** | **28.58°** | **6.43** | **15.98°** | **41.18°** | **-2.13** | 25.57 | 22.71 | 17.22 | 28.20 | 0.13 |
| 1989 | Breeding | 25.33° | 28.44° | 6.77 | 15.17° | 41.71° | -0.46 | 25.76 | 25.25 | 19.47 | 31.03 | 0.02 |
| 1990 | Breeding | **16.93°** | **30.62°** | **5.42** | **20.00°** | **41.24°** | **-2.53** | 29.13 | 26.32 | 20.97 | 31.67 | 0.11 |
| 1991 | Breeding | **15.86°** | **32.11°** | **5.31** | **21.70°** | **42.52°** | **-3.09** | 32.21 | 28.44 | 23.07 | 33.81 | 0.13 |
| 1992 | Breeding | **12.54°** | **33.71°** | **5.16** | **23.60°** | **43.82°** | **-4.10** | **36.05** | **29.06** | **23.47** | **34.65** | **0.24** |
| 1993 | Breeding | **18.67°** | **33.39°** | **7.00** | **19.67°** | **47.11°** | **-2.10** | **39.79** | **28.09** | **21.94** | **34.24** | **0.42** |
| 1994 | Breeding | **8.52°** | **33.37°** | **4.96** | **23.65°** | **43.09°** | **-5.01** | **42.14** | **29.58** | **23.86** | **35.30** | **0.42** |
| 1995 | Breeding | **10.66°** | **34.35°** | **5.02** | **24.51°** | **44.19°** | **-4.72** | **41.21** | **29.57** | **24.00** | **35.14** | **0.39** |
| 1996 | Breeding | **10.46°** | **33.42°** | **4.94** | **23.75°** | **43.11°** | **-4.65** | **41.19** | **29.39** | **23.75** | **35.03** | **0.40** |
| 1997 | Breeding | **10.88°** | **35.74°** | **4.83** | **26.27°** | **45.21°** | **-5.15** | **47.35** | **32.51** | **26.90** | **38.12** | **0.46** |
| 1998 | Breeding | **17.58°** | **37.47°** | **5.24** | **27.20°** | **47.74°** | **-3.80** | **37.75** | **28.99** | **23.60** | **34.38** | **0.30** |
| 1999 | Breeding | **10.66°** | **34.99°** | **5.89** | **23.45°** | **46.53°** | **-4.13** | **38.58** | **27.97** | **21.64** | **34.30** | **0.38** |
| 2000 | Breeding | **9.80°** | **36.66°** | **4.76** | **27.33°** | **45.99°** | **-5.64** | **48.53** | **34.80** | **29.00** | **40.60** | **0.39** |
| 2001 | Breeding | **12.51°** | **37.95°** | **4.95** | **28.25°** | **47.65°** | **-5.14** | **41.75** | **30.20** | **24.59** | **35.61** | **0.38** |
| 2002 | Breeding | **14.56°** | **41.77°** | **5.09** | **31.79°** | **51.75°** | **-5.35** | **39.95** | **31.67** | **26.28** | **37.06** | **0.26** |
| 2003 | Breeding | **15.73°** | **39.68°** | **5.12** | **29.64°** | **49.72°** | **-4.68** | **46.97** | **33.07** | **27.66** | **38.48** | **0.42** |
| 2004 | Breeding | **16.56°** | **40.02°** | **4.98** | **30.26°** | **49.78°** | **-4.71** | **49.53** | **35.76** | **30.13** | **41.39** | **0.39** |
| 2005 | Breeding | **16.01°** | **40.00°** | **4.99** | **30.22°** | **49.78°** | **-4.81** | **49.27** | **36.79** | **31.24** | **42.34** | **0.34** |
| 2006 | Breeding | **12.01°** | **38.29°** | **4.81** | **28.86°** | **47.72°** | **-5.46** | **50.07** | **37.35** | **31.92** | **42.78** | **0.34** |
| 2007 | Breeding | **15.34°** | **41.80°** | **4.43** | **33.12°** | **50.48°** | **-5.97** | **53.75** | **40.00** | **34.73** | **45.27** | **0.34** |
| 2008 | Breeding | **17.54°** | **43.22°** | **4.48** | **34.44°** | **52.00°** | **-5.73** | **54.09** | **40.40** | **35.26** | **45.54** | **0.34** |
| 2009 | Breeding | **16.89°** | **43.65°** | **4.53** | **34.77°** | **52.53°** | **-5.91** | **53.17** | **41.54** | **36.25** | **46.83** | **0.28** |
| 2010 | Breeding | **17.07°** | **42.15°** | **4.73** | **32.88°** | **51.42°** | **-5.30** | **52.80** | **40.51** | **35.18** | **45.84** | **0.30** |
| 2011 | Breeding | **15.64°** | **44.06°** | **4.29** | **35.65°** | **52.47°** | **-6.62** | **55.68** | **41.65** | **36.59** | **46.71** | **0.34** |
| 1984 | Wintering | **4.20°** | **24.67°** | **5.61** | **13.67°** | **35.67°** | **-3.65** | **17.75** | **13.34** | **9.09** | **17.59** | **0.33** |
| 1985 | Wintering | **13.59°** | **32.51°** | **5.40** | **21.93°** | **43.09°** | **-3.50** | **31.33** | **24.17** | **19.52** | **28.82** | **0.30** |
| 1986 | Wintering | No convergence attained | | | | | | **16.52** | **10.26** | **6.12** | **14.40** | **0.58** |
| 1987 | Wintering | 20.24° | 26.28° | 6.94 | 12.68° | 39.88° | -0.87 | 18.58 | 16.28 | 12.28 | 20.28 | 0.14 |
| 1988 | Wintering | 18.74° | 28.49° | 7.24 | 14.30° | 42.68° | -1.35 | 18.84 | 20.47 | 15.82 | 25.12 | -0.08 |
| 1989 | Wintering | **21.84°** | **35.14°** | **5.15** | **25.05°** | **45.23°** | **-2.58** | 26.28 | 25.97 | 21.76 | 30.18 | 0.01 |
| 1990 | Wintering | **18.35°** | **33.26°** | **4.98** | **23.50°** | **43.02°** | **-2.99** | 25.15 | 24.05 | 19.84 | 28.26 | 0.05 |
| 1991 | Wintering | **17.66°** | **38.96°** | **4.18** | **30.77°** | **47.15°** | **-5.10** | **37.62** | **33.35** | **29.51** | **37.19** | **0.13** |
| 1992 | Wintering | **15.09°** | **37.48°** | **4.27** | **29.11°** | **45.85°** | **-5.24** | **36.69** | **30.23** | **25.96** | **34.50** | **0.21** |
| 1993 | Wintering | **19.69°** | **37.84°** | **4.23** | **29.55°** | **46.13°** | **-4.29** | 31.49 | 30.64 | 26.50 | 34.78 | 0.03 |
| 1994 | Wintering | **13.05°** | **33.18°** | **4.81** | **23.75°** | **42.61°** | **-4.19** | **34.58** | **27.84** | **23.27** | **32.41** | **0.24** |
| 1995 | Wintering | **20.53°** | **35.62°** | **4.46** | **26.88°** | **44.36°** | **-3.38** | **36.87** | **31.24** | **27.01** | **35.47** | **0.18** |
| 1996 | Wintering | **14.41°** | **35.76°** | **4.73** | **26.49°** | **45.03°** | **-4.51** | **39.74** | **30.77** | **26.18** | **35.36** | **0.29** |
| 1997 | Wintering | **14.76°** | **38.50°** | **4.21** | **30.25°** | **46.75°** | **-5.64** | **44.02** | **33.54** | **29.40** | **37.68** | **0.31** |
| 1998 | Wintering | **15.83°** | **33.50°** | **4.47** | **24.74°** | **42.26°** | **-3.95** | 28.59 | 25.57 | 21.51 | 29.63 | 0.12 |
| 1999 | Wintering | **21.74°** | **43.31°** | **4.40** | **34.69°** | **51.93°** | **-4.90** | 36.10 | 32.29 | 28.12 | 36.46 | 0.12 |
| 2000 | Wintering | **15.27°** | **41.08°** | **4.00** | **33.24°** | **48.92°** | **-6.45** | **44.37** | **35.62** | **31.29** | **39.95** | **0.25** |
| 2001 | Wintering | **11.31°** | **38.42°** | **4.32** | **29.95°** | **46.89°** | **-6.28** | **44.40** | **31.63** | **27.16** | **36.10** | **0.40** |
| 2002 | Wintering | **13.99°** | **39.04** | **4.28** | **30.65°** | **47.43°** | **-5.85** | **40.89** | **32.73** | **28.18** | **37.28** | **0.25** |
| 2003 | Wintering | **16.28°** | **41.47°** | **4.27** | **33.10°** | **49.84°** | **-5.90** | **47.86** | **35.36** | **30.89** | **39.83** | **0.35** |
| 2004 | Wintering | **16.88°** | **41.30°** | **4.19** | **33.09°** | **49.51°** | **-5.83** | **52.85** | **33.11** | **28.72** | **37.50** | **0.60** |
| 2005 | Wintering | **18.20°** | **46.64°** | **3.70** | **39.39°** | **53.89°** | **-7.69** | **61.62** | **41.72** | **37.68** | **45.76** | **0.48** |
| 2006 | Wintering | **14.60°** | **45.85°** | **3.79** | **38.42°** | **53,28°** | **-8.25** | **62.08** | **41.21** | **37.09** | **45.33** | **0.51** |
| 2007 | Wintering | **14.22°** | **45.00°** | **3.89** | **37.38°** | **52.62°** | **-7.91** | **61.35** | **38.84** | **34.47** | **43.21** | **0.58** |
| 2008 | Wintering | **16.20°** | **46.12°** | **3.42** | **39.42°** | **52.82°** | **-8.75** | **63.00** | **43.36** | **39.07** | **47.65** | **0.45** |
| 2009 | Wintering | **11.31°** | **43.65°** | **3.68** | **36.44°** | **50.86°** | **-8.79** | **69.27** | **42.47** | **38.12** | **46.82** | **0.63** |
| 2010 | Wintering | **12.35°** | **43.89°** | **3.67** | **36.70°** | **51.08°** | **-8.59** | **30.56** | **39.97** | **35.80** | **44.14** | **0.52** |
| 2011 | Wintering | **16.75°** | **42.12°** | **4.12** | **34.03°** | **50.20°** | **-6.16** | **56.59** | **37.08** | **32.71** | **41.45** | **0.53** |
| Quantitative matrix | | | | | | | | | | | | |
| WNODF | | | | | | | | | | | | |
| Year | Season | Observed | Simulated | SD | Lower  95 % CI | Upper  95 % CI | Z' | | | | | |
| 1984 | Breeding | **7.84** | **15.52** | **1.11** | **13.12** | **17.35** | **-6.95** | | | | | |
| 1985 | Breeding | **15.86** | **34.07** | **1.75** | **30.17** | **37.13** | **-10.43** | | | | | |
| 1986 | Breeding | **14.42** | **27.76** | **1.45** | **24.66** | **30.23** | **-9.18** | | | | | |
| 1987 | Breeding | **13.72** | **24.19** | **1.55** | **20.54** | **26.80** | **-6.74** | | | | | |
| 1988 | Breeding | **15.68** | **28.75** | **1.33** | **25.91** | **31.17** | **-9.82** | | | | | |
| 1989 | Breeding | **15.20** | **27.83** | **1.35** | **25.05** | **30.30** | **-9.32** | | | | | |
| 1990 | Breeding | **17.09** | **36.00** | **1.48** | **33.06** | **38.81** | **-12.76** | | | | | |
| 1991 | Breeding | **20.19** | **37.36** | **1.58** | **34.21** | **40.11** | **-10.88** | | | | | |
| 1992 | Breeding | **24.90** | **40.51** | **1.73** | **36.86** | **43.39** | **-9.02** | | | | | |
| 1993 | Breeding | **26.99** | **33.87** | **1.90** | **30.18** | **37.56** | **-3.63** | | | | | |
| 1994 | Breeding | **24.45** | **47.22** | **2.32** | **41.90** | **51.23** | **-9.83** | | | | | |
| 1995 | Breeding | **24.02** | **47.57** | **2.69** | **41.83** | **52.23** | **-8.74** | | | | | |
| 1996 | Breeding | **23.67** | **46.24** | **1.99** | **42.09** | **49.69** | **-11.33** | | | | | |
| 1997 | Breeding | **28.67** | **57.65** | **2.39** | **52.77** | **62.19** | **-12.13** | | | | | |
| 1998 | Breeding | **18.54** | **49.87** | **2.38** | **44.65** | **53.77** | **-13.17** | | | | | |
| 1999 | Breeding | **16.29** | **35.55** | **1.94** | **31.28** | **38.76** | **-9.93** | | | | | |
| 2000 | Breeding | **23.75** | **52.21** | **2.68** | **46.86** | **56.93** | **-10.61** | | | | | |
| 2001 | Breeding | **17.92** | **47.66** | **2.19** | **43.10** | **51.62** | **-13.59** | | | | | |
| 2002 | Breeding | **18.57** | **55.28** | **2.00** | **50.82** | **58.83** | **-18.36** | | | | | |
| 2003 | Breeding | **23.88** | **51.94** | **2.70** | **45.75** | **56.41** | **-10.38** | | | | | |
| 2004 | Breeding | **24.98** | **55.80** | **2.75** | **49.53** | **60.49** | **-11.19** | | | | | |
| 2005 | Breeding | **25.02** | **55.81** | **1.88** | **51.81** | **59.20** | **-16.39** | | | | | |
| 2006 | Breeding | **27.37** | **55.56** | **2.57** | **50.18** | **59.96** | **-10.95** | | | | | |
| 2007 | Breeding | **27.72** | **60.98** | **2.88** | **55.22** | **65.70** | **-11.56** | | | | | |
| 2008 | Breeding | **26.02** | **61.97** | **2.51** | **56.64** | **66.57** | **-14.32** | | | | | |
| 2009 | Breeding | **27.28** | **60.48** | **1.91** | **56.58** | **63.94** | **-17.34** | | | | | |
| 2010 | Breeding | **25.77** | **60.43** | **2.03** | **55.94** | **63.97** | **-17.07** | | | | | |
| 2011 | Breeding | **30.69** | **65.79** | **2.32** | **61.01** | **69.81** | **-15.10** | | | | | |
| 1984 | Wintering | **13.07** | **22.57** | **1.29** | **19.91** | **24.84** | **-7.37** | | | | | |
| 1985 | Wintering | **22.09** | **36.90** | **1.23** | **34.28** | **39.05** | **-12.06** | | | | | |
| 1986 | Wintering | **9.81** | **18.62** | **1.26** | **15.85** | **20.72** | **-6.99** | | | | | |
| 1987 | Wintering | **11.73** | **25.16** | **0.97** | **23.18** | **26.97** | **-13.82** | | | | | |
| 1988 | Wintering | **12.09** | **20.61** | **0.86** | **18.75** | **22.12** | **-9.94** | | | | | |
| 1989 | Wintering | **16.90** | **30.19** | **1.12** | **27.89** | **32.08** | **-11.88** | | | | | |
| 1990 | Wintering | **16.35** | **30.16** | **1.08** | **27.85** | **32.03** | **-12.79** | | | | | |
| 1991 | Wintering | **22.34** | **47.44** | **1.65** | **43.94** | **50.39** | **-15.24** | | | | | |
| 1992 | Wintering | **24.53** | **46.99** | **1.43** | **44.27** | **49.60** | **-15.65** | | | | | |
| 1993 | Wintering | **21.45** | **44.73** | **1.35** | **42.03** | **47.17** | **-17.22** | | | | | |
| 1994 | Wintering | **22.54** | **43.07** | **1.63** | **39.76** | **45.90** | **-12.59** | | | | | |
| 1995 | Wintering | **25.44** | **48.33** | **1.51** | **44.86** | **50.86** | **-15.19** | | | | | |
| 1996 | Wintering | **28.05** | **50.06** | **1.66** | **46.47** | **53.06** | **-13.22** | | | | | |
| 1997 | Wintering | **28.43** | **52.29** | **1.60** | **48.99** | **55.14** | **-14.88** | | | | | |
| 1998 | Wintering | **18.64** | **41.77** | **1.48** | **38.45** | **44.30** | **-15.60** | | | | | |
| 1999 | Wintering | **19.92** | **49.53** | **1.76** | **45.92** | **52.71** | **-16.87** | | | | | |
| 2000 | Wintering | **26.26** | **51.47** | **1.38** | **48.51** | **53.84** | **-18.31** | | | | | |
| 2001 | Wintering | **30.22** | **53.10** | **2.12** | **48.77** | **57.02** | **-10.78** | | | | | |
| 2002 | Wintering | **25.82** | **46.03** | **1.63** | **42.58** | **48.85** | **-12.42** | | | | | |
| 2003 | Wintering | **31.15** | **54.31** | **1.80** | **50.04** | **57.43** | **-12.85** | | | | | |
| 2004 | Wintering | **34.42** | **63.35** | **2.29** | **58.50** | **67.59** | **-12.61** | | | | | |
| 2005 | Wintering | **39.17** | **69.11** | **1.99** | **65.15** | **72.69** | **-15.08** | | | | | |
| 2006 | Wintering | **38.72** | **69.17** | **2.13** | **64.97** | **73.18** | **-14.31** | | | | | |
| 2007 | Wintering | **40.17** | **68.47** | **2.15** | **63.82** | **72.27** | **-13.19** | | | | | |
| 2008 | Wintering | **41.80** | **69.35** | **1.90** | **65.50** | **72.90** | **-14.49** | | | | | |
| 2009 | Wintering | **45.52** | **70.96** | **1.93** | **66.99** | **74.49** | **-13.16** | | | | | |
| 2010 | Wintering | **40.99** | **67.41** | **2.01** | **63.26** | **70.98** | **-13.13** | | | | | |
| 2011 | Wintering | **37.20** | **60.85** | **1.70** | **57.44** | **64.05** | **-13.94** | | | | | |
