# Supplementary material for: A Long-Term Macroecological Analysis of the Recovery of a Waterbird Metacommunity after Site Protection
Source: PLoS One. 2014 Aug 18;9(8):e105202. doi: 10.1371/journal.pone.0105202 (PMC4136829; doi:10.1371/journal.pone.0105202)
Supplement: Table S2 — Order of nestedness of the overall qualitative matrix for breeding and wintering season. The order of nestedness is according to the degree of nestedness packed by BINMATNEST. (DOCX) [file pone.0105202.s003.docx]

| Breeding | | | Wintering | | | |
| --- | --- | --- | --- | --- | --- | --- |
| Order | Site | # species | Order | Site | # species |  |
| 1 | P. N. Salinas de Santa Pola | 38 | 1 | P. N. de L´Albufera | 64 |  |
| 2 | P. N. de El Hondo | 37 | 2 | Marjal de Pego-Oliva | 62 |  |
| 3 | P. N. de l‘Albufera | 36 | 3 | P. N. Salinas de Santa Pola | 59 |  |
| 4 | Marjal de Almenara | 30 | 4 | Marjal de Almenara | 54 |  |
| 5 | Marjal de Pego-Oliva | 28 | 5 | Marjal del Moro | 55 |  |
| 6 | Marjal del Moro | 27 | 6 | P. N. de El Hondo | 55 |  |
| 7 | Marjal de Xeresa-Xeraco | 26 | 7 | Laguna Mata-Torrevieja | 51 |  |
| 8 | Laguna de La Mata-Torrevieja | 21 | 8 | Marjal de Xeresa-Xeraco | 52 |  |
| 9 | Clot de Galvany/Balsares | 21 | 9 | Delta del Mijares | 43 |  |
| 10 | Prat de Cabanes-Torreblanca | 20 | 10 | Prat de Cabanes-Torreblanca | 37 |  |
| 11 | Embalse de La Pedrera | 20 | 11 | Barranc del Carraixet | 38 |  |
| 12 | Hondo de Amorós | 15 | 12 | Clot de Galvany/Balsares | 41 |  |
| 13 | Embalse de Elche | 13 | 13 | Desembocadura del Segura | 36 |  |
| 14 | Saladar de Agua Amarga | 11 | 14 | Embalse de La Pedrera | 34 |  |
| 15 | Delta del Mijares | 10 | 15 | Hondo de Amorós | 30 |  |
| 16 | Desembocadura del Segura | 9 | 16 | Saladar de Agua Amarga | 30 |  |
| 17 | Salinas de Calpe | 6 | 17 | Salinas de Calpe | 27 |  |
| 18 | Barranc del Carraixet | 5 | 18 | Embalse de Elche | 25 |  |
